# LPS Mediates Bovine Endometrial Epithelial Cell Pyroptosis Directly Through Both NLRP3 Classical and Non-Classical Inflammasome Pathways

**DOI:** 10.3389/fimmu.2021.676088

**Published:** 2021-05-28

**Authors:** Xiaoyu Ma, Yajuan Li, Wenxiang Shen, Ayodele Olaolu Oladejo, Jie Yang, Wei Jiang, Bereket Habte Imam, Xiaohu Wu, Xuezhi Ding, Ying Yang, Shengyi Wang, Zuoting Yan

**Affiliations:** ^1^ Key Laboratory of Veterinary Pharmaceutical Development, Ministry of Agricultural and Rural Affairs, Lanzhou Institute of Husbandry and Pharmaceutical Science, Chinese Academy of Agricultural Science, Lanzhou, China; ^2^ College of Veterinary Medicine, Inner Mongolia Agricultural University, Hohhot, China; ^3^ Department of Animal Health Technology, Oyo State College of Agriculture and Technology, Igboora, Nigeria

**Keywords:** BEEC, LPS, inflammasome, GSDMD, pyroptosis

## Abstract

As a highly inflammatory form of programmed cell death, pyroptosis is triggered by pro-inflammatory signals and associated with inflammation. It is characterized by cell swelling and large bubbles emerging from the plasma membrane, which release cytokines during inflammation. Compared with other types of cell death, pyroptosis has a distinct morphology and mechanism and involves special inflammasome cascade pathways. However, the inflammasome mechanism through which endometrial epithelial cell pyroptosis occurs in LPS-mediated inflammation remains unclear. We confirmed that there was an increased mRNA and protein expression of the IL-6, TNF-α, IL-1β, IL-18 cytokines, the inflammasome molecules NLRP3, CASPASE-1, CASPASE-4, and GSDMD in LPS-induced primary bovine endometrial epithelial cells (BEECs) in an *in vitro* established inflammatory model using ELISA, real-time PCR (RT-PCR), vector construction and transfection, and Western blotting. Scanning electron microscopy and lactate dehydrogenase (LDH) activity assays revealed induced cell membrane rupture, which is the main characteristic of pyroptosis. In conclusion, the cytolytic substrate GSDMD’s cleavage by caspase-1 or caspase-4 through the NLRP3 classical and non-classical inflammasome pathways, GSDMD N-terminus bind to the plasma membrane to form pores and release IL -18, IL-1β cause cell death during LPS induced BEECs inflammation.

## Introduction

The uterus is the organ for embryonic development and the supply of fetal nutrition through the feto-maternal interactions. It is also an organ with a complex immune repertoire. The complete tissue structure and healthy function of the uterus are the basis of maternal reproduction. Almost all cows’ reproductive systems will be infected with bacteria after parturition (1, 2). The inflammatory environment is increased in the bovine uterus due to bacterial contamination and tissue degradation driven by local pathogen-associated molecular patterns (PAMPs) and damage-associated molecular patterns (DAMPs). Post-parturition inflammation has been described as a normal physiological event, indicating that the bovine uterus needs proper tissue remodeling to restore its homeostasis for future pregnancy (3, 4). Although, most cows try to eliminate bacterial contamination in the uterus during post parturient through the uterine mucosal immune response, about half of postpartum cows still develop endometritis (5), which has a high incidence of disease in postpartum cows worldwide. Reports have shown that clinical endometritis occurs in more than 40% of cows within first three weeks postpartum (6), with its 67% occurring in Europe and North America (7). Research evidences have shown that high-yield cows have more susceptible to the disease than low-yield cows (8). Cow endometritis induces uterine tissue damage, embryonic death, a prolonged luteal phase, and increased fetal distance, infertility, and fetal death (9, 10), and directly reduces the economic value of dairy cows, retards the dairy cattle breeding development, which causes great harm to postpartum dairy cows’ health recovery and serious losses in the dairy industry. Some cattle also develop sub-clinical endometritis partially due to persistent uterine infection (11, 12), making it even more difficult to cure and resulting in a higher economic loss (13).

Pathogenic microorganisms, such as bacteria, play an important role in the pathogenesis of endometritis in dairy cows, including the *Escherichia coli*, *Trueprella pyogenes*, *Prevotella species*, and *Streptococcus pyogenes. E. coli* and *S. pyogenes* are the most widespread pathogens, and the uterus is most susceptible to *E. coli* infection (14–16). Etiological studies have shown that infection of the female genital tract by Gram-negative bacteria is one of the most important causes of infertility, early abortion, and uterine infection. The lipopolysaccharide (LPS) endotoxin, a PAMP produced by Gram-negative bacteria, is the fundamental factor that triggers the immune response associated with endometritis (17–19).

Pyroptosis, a type of inflammatory necrosis, is a highly inflammatory form of programmed cell death (20), characterized by cell swelling and large bubbles emerging from the plasma membrane (21, 22). Pyroptosis has a distinct morphology and mechanism as compared to other types of cell death (23–25). Caspase-1, an interleukin-1 converting enzyme, proteolytically cleaves the precursors of the inflammatory cytokines, such as IL-1β and IL-18, as well as the pyroptosis inducer gasdermin D (GSDMD) (26) to trigger pyroptosis *via* the classical inflammasome pathway (27, 28). Pro-caspase-1 can be recruited and activated within inflammasomes following its assembly (29). Other studies have shown that another means of mediating pyroptosis, caspase-11 in mice, or caspase-4/5 in humans, can serve as direct receptors for LPS. After binding with LPS, it cleaves GSDMD directly, leading to pyroptosis *via* the non-classical inflammasome pathway (30–32). NACHT, leucine-rich repeat sequence (LRR), and PYRIN-PAAD-DAPIN (PYD) domains-containing protein 3 (NLRP3), also known as NALP3 and cryopyrin, belongs to the NOD-like receptor (NLR) family, together with the adaptor apoptosis-associated speck-like protein containing CARD (ASC) protein, and PYDCARD, composed of the N-terminal PYD domain protein and a C-terminal caspase-recruitment domain (CARD) protein, form the caspase-1 activating complex known as the NLRP3 inflammasome (26). Increasing evidence indicates that the NLRP3 inflammasome, the most characterized and studied inflammasome, responds to various activators, such as microorganisms and their derived products, and endogenous danger signals (29, 33, 34).

Research on pyroptosis appeared early, but pyroptosis has often confused with apoptosis. The mechanism by which inflammasome mediates pyroptosis was found in the Shao Feng laboratory and the Vishva Dixit laboratory in 2015 (23, 24). Their research showed that cell pyroptosis was caused by the cleavage of GSDMD protein, causing its N-terminal part to oligomerize on the cell membrane and trigger pores, causing the cell membrane to rupture.

Pyroptosis induced by the inflammasome pathways could be one of the molecular mechanisms in dairy cow endometritis pathogenesis. Therefore, this study aims to investigate which inflammasome (classical or non-classical) pathway, or both, plays a role in molecular cell death during endometritis. We hypothesized that pyroptosis in bovine endometritis could occur through the NLRP3 classical or non-classical inflammasome pathways or both.

## Materials and Methods

### Reagents and Antibodies

Dulbecco’s Modified Eagle Medium (DMEM, high glucose) and phosphate buffer solution (PBS) were purchased from Hyclone (Logan, Utah, USA). Fetal bovine serum (FBS) was purchased from Gibco (Grand Island, USA). Trypsin and Lipopolysaccharide (LPS, *E. coli* O111: B4) were purchased from Sigma (USA). The LDH assay kit was purchased from Nanjing Jiancheng Bioengineering Institute (Nanjing, China). Bovine IL-6, IL-1β, and TNF-α ELISA Kits and the 2.5% glutaraldehyde (EM Grade), were purchased from Solarbio (Beijing, China). TRIzol™ Reagent was purchased from Invitrogen (Carlsbad, California, USA). The Evo M-MLV RT-PCR Kit and SYBR^®^ Green Premix Pro Taq HS qPCR Kit were purchased from Accurate Biotechnology (Hunan, China). The ProteinExt^®^ Mammalian Total Protein Extraction Kit was purchased from TransGen Biotech (Beijing, China). The BCA Protein Assay kit was purchased from Takara (Dalian, China). Rabbit anti‐bovine NLRP3 antibody (19771-1-AP), rabbit anti‐bovine ASC antibody (10500-1-AP), and rabbit anti‐bovine caspase-1 antibody (22915-1-AP) were purchased from Protein Tech Group (Chicago, USA). Rabbit anti‐bovine caspase-4 antibody (GTX86890) was purchased from Gene Tex (Southern California, USA). Anti-beta actin antibody (ab8226) and Anti-HA tag antibody (ab9110) were purchased from Abcam (Cambridge, MA, USA). Odyssey^®^ Blocking Buffer (TBS), IRDye^®^ 800 CW goat anti-mouse IgG, and IRDye^®^ 680 RD goat anti-rabbit IgG were purchased from LI-COR (Lincoln City, Nebraska, USA). The pCMV-GSDMD-HA vector was constructed by Genecreate Biological (Wuhan, China), LB Broth and LB Nutrient Agar were purchased from Hope Bio-Technology (Qingdao, China), Ampicillin sodium was purchased from Sangon Biotech (Shanghai, China), and Endo-free Plasmid Mini Kit I was purchased from Omega (USA). Zeta Transfection Kit was purchased from Zeta Life (USA).

### Cell Culture

Bovine endometrial epithelial cells (BEECs) were isolated from a healthy uterus of a six-month-old dairy cow by our laboratory (35) and were cultured in DMEM supplemented with 10% FBS at 37°C in a humidified atmosphere with 5% CO_2_. LPS (1 mg) was diluted into 0, 3, 10, and 30 µg/ml by DMEM without FBS. The cell concentration was adjusted to 2 × 10^5^/ml, and the cells were cultured in the 6-well plate for 24 h; the supernatant was then exchanged replaced with new DMEM containing different concentrations of LPS and 10% FBS, and cultured for an additional 24 h. Each group was conducted in triplicates.

### LDH Assay and Cytokine Detection

Cultured the BEECs normally and adjusted the density to 2 × 10^5^/ml. Then transferred cells to the 6-well plate and incubated at 37°C with 5% CO_2_ for 24 h. After exposure with different concentrations of LPS for 24 h, and each concentration repeated three times, took out the 6-well plate from the incubator, then the extracellular fluid of each group was collected and centrifuged as required for the kits, and the supernatant was used for the LDH activity assay and cytokine content (IL-6, IL-1β, and TNF-α) analysis *via* ELISA.

### Scanning Electron Microscopy

The BEECs was reactivated and adjusted the density to 2 × 10^5^/ml, transferred cells to the 6-well plate, which contains a 25 mm^2^ cell slide in each well, and incubated at 37°C with 5% CO_2_ for 24 h. The cells were observed to grow normally on the slides under an inverted microscope. After exposure to different concentrations of LPS for 24 h in the incubator, the cultured cells were taken out and washed twice with 2 ml PBS, then added 3 ml 2.5% glutaraldehyde, and incubated at room temperature for 1 hour then stored at 4°C. This was followed up by processing, scanning electron microscope observation and photographing (Lilai Biotechnology Company, Chengdu, China).

### Quantitative Real-Time PCR

**Figure 1:**
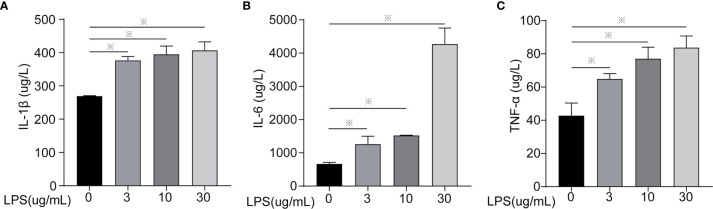
Inflammatory cytokines content of cells treated with different concentrations of LPS for 24 h. **(A)** IL-1β, **(B)** IL-6, and **(C)** TNF-α. Each treatment had three samples (n = 3) and was repeated three times to reduce system errors; “※” means there was a significant difference (*P < 0.05*) compared with negative control (LPS, 0 µg/ml).

Total RNA from the experimented cells was extracted using TRIzol™ Reagent and quantified using a Nanodrop spectrophotometer (Pultton P100+ type) and RNA gel electrophoresis (**Supplemental Table 1** and **Figure 1**). cDNA was produced by reverse-transcribing the isolated total RNA using an Evo M-MLV RT for PCR Kit and Biometra TOne 96 G PCR machine (Biometra GmbH). The primers are presented in **Supplemental Table 2**. The qPCR was analyzed using the SYBR^®^ Green Premix Pro Taq HS qPCR Kit, and the gene expression levels were measured by reverse transcription and RT-qPCR (Quantstudio™ 5 Real-Time PCR system, Applied Biosystems, Singapore) and quantified using the 2^−ΔΔCt^ method (36). Three technical replicates were used for all reactions.

### Western Blotting

Total proteins were extracted using the ProteinExt^®^ Mammalian Total Protein Extraction Kit and quantified with the BCA Protein Assay Kit and a Multiskan™ GO Full-wavelength microplate reader (Thermo Fisher Scientific) at a wavelength of 562 nm. The protein concentration of each group was adjusted with sterile water according to the results of the BCA assay, 6 × Loading Buffer was added and then placed in a metal bath at 100°C for 5–15 min to denature the protein. Separated *via* 10% sodium dodecyl sulfate-polyacrylamide gel electrophoresis (SDS-PAGE), then transferred onto NC membranes. Odyssey^®^ Blocking Buffer (TBS) was added and samples were incubated 1 h at room temperature, then incubated with primary antibodies overnight at 4°C. The membranes were washed and then incubated with secondary antibodies for 2 h at room temperature, and then washed again, and the target bands were visualized using the Odyssey^®^ CLx Imaging System (LI-COR, USA).

### Genetic Recombination

Genetic Recombination Technology was adopted to ligate bovine GSDMD DNA fragments into the ampicillin-resistant pCMV-HA vector and transform it into *E. coli.* Briefly, the bacteria were grown on an LB agar medium containing ampicillin sodium for 24 h at 37°C. A single colony was picked and added to the LB liquid medium containing ampicillin sodium and cultured for 16 h at 37°C with shaking at 300×*g*. Plasmid DNA was extracted with an Endo-free Plasmid Mini Kit I and quantified using a Polluton100+. The Zeta Transfection Kit was used to transfect the plasmid into BEECs, and LPS was used to construct the cell inflammation model. The cell total protein was extracted and detected by an HA tag and Western blotting to evaluate the GSDMD protein expression and its cleavage during cell pyroptosis. The vector construction, restriction endonuclease cleavage verification, and sequencing verification were done by Genecreate Biological Company (Wuhan, China).

### Statistical Analysis

The data were analyzed using the SPSS 20.0 software (IBM). Normally distributed data from different groups were compared by one-way ANOVA with *P <0.05* being considered statistically significant. Graph Pad 8 software (GraphPad Prism San Diego, CA, USA), Adobe Photoshop 2021, and Adobe Illustrator 2019 were used for image creation.

## Results

### LPS Induced BEEC Inflammatory Response

**Figure 2:**
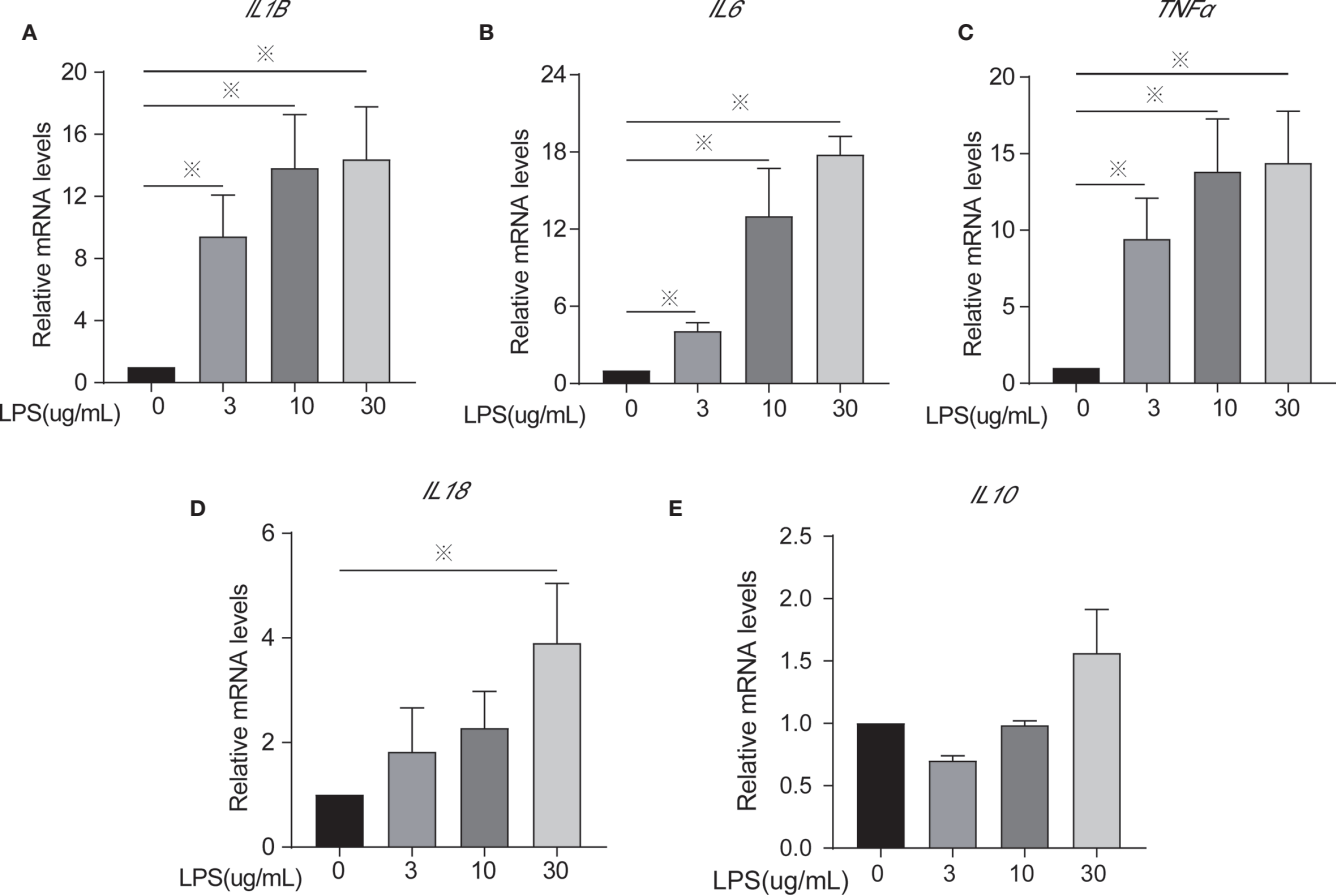
Inflammatory cytokines mRNA expression of cells treated with different concentrations of LPS for 24 h. **(A)** *IL1β*, **(B)** *IL6*, **(C)** *TNFα*, **(D)** *IL18*, and **(E)** *IL10*. Each treatment had three samples (n = 3) and was repeated three times to reduce system errors; “※” means there was a significant difference (*P < 0.05*) compared with negative control (LPS, 0 µg/ml).

Inflammatory factors (IL-6, IL-1β, TNF-α, IL-18, and IL-10) were measured to identify the degree of cell inflammation (**Figures 1** and **2**). After LPS exposure for 24 h, the treated groups (LPS, 3, 10, 30 µg/ml) had significantly increased IL-6, IL-1β, and TNF-α contents in the extracellular fluid (**Figure 1**) and mRNA expression (**Figures 2A–C**), compared to controls (LPS, 0 µg/ml). Only the 30 µg/ml group had significant *IL18* mRNA expression (**Figure 2D**). No statistically significant difference was seen for *IL10* mRNA expression among treatments (**Figure 2E**).

### LPS Induced BEEC Cell Membrane Rupture

**Figure 3:**
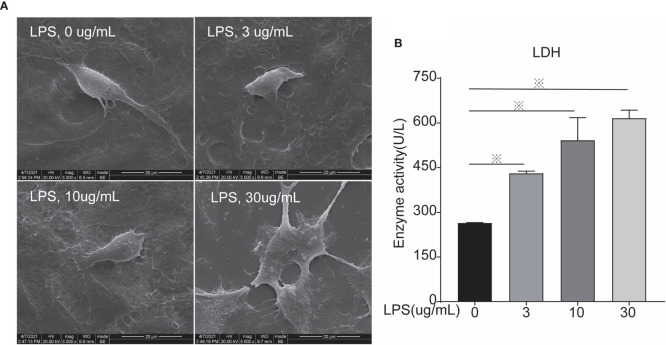
Scanning Electron Microscopy and LDH activity of cells treated with different concentrations of LPS for 24 h. **(A)** Scanning electron microscopy, and **(B)** LDH. Each treatment had three samples (n = 3) and was repeated three times to reduce system errors; “※” means there was a significant difference (*P < 0.05*) compared with negative control (LPS, 0 µg/ml); scanning electron microscopy photographing magnification is 5,000 times, and the picture scale was 20 um.

Morphological changes of cells were analyzed using scanning electron microscopy after 24 h of LPS exposure. In the control group, the morphology was normal and the cell membrane was intact. The cell morphology of the 3 µg/ml group was still normal, whereas the cell membrane was ruptured in the 10 µg ml group. In the 30 µg/ml group the cell shape was unclear because the cell membrane was largely damaged, suggesting cell death (**Figure 3A**). LDH activity was measured to evaluate cell mortality (**Figure 3B**). After LPS exposure for 24 h, the treated groups (LPS, 3, 10, 30 µg/ml) had significantly increase LDH activity compared to controls (LPS, 0 µg/ml), leading to higher cell mortality *via* cell membrane rupture.

### LPS Exposure for 24 h Leads to BEEC Inflammation *via* Inflammasomes

**Figure 4:**
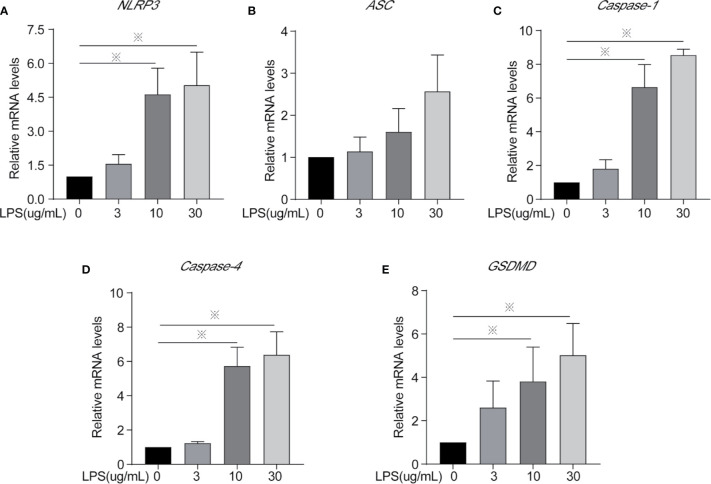
Inflammasomes mRNA expression of cells treated with different concentrations of LPS for 24 h. **(A)** *NLRP3*, **(B)** *ASC*, **(C)** *Caspase-1*, **(D)** *Caspase-4*, and **(E)** *GSDMD*. Each treatment had three samples (n = 3) and was repeated three times to reduce system errors; “※” means there was a significant difference (*P < 0.05*) compared with negative control (LPS, 0 µg/ml).

**Figure 5:**
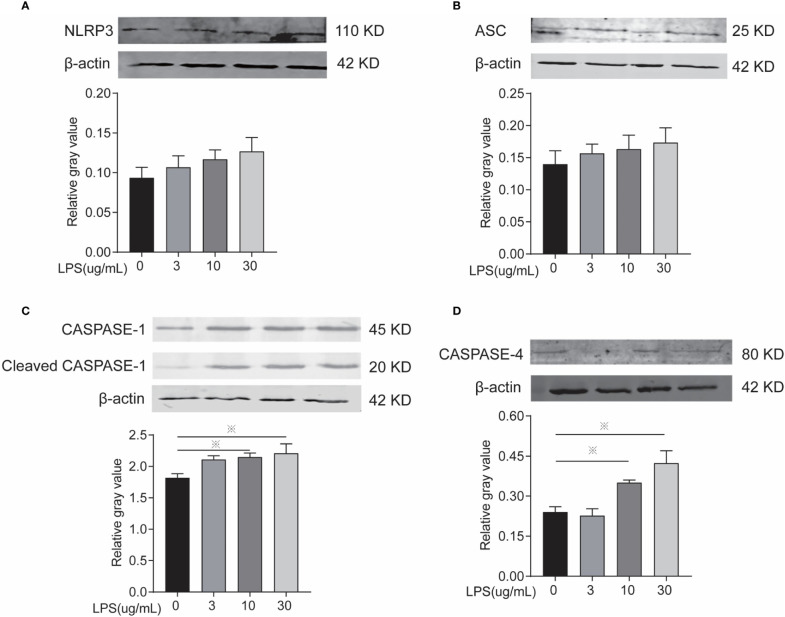
Inflammasomes protein expression of cells treated with different concentrations of LPS for 24 h. **(A)** Protein electrophoresis, **(B)** NLRP3, **(C)** ASC, **(D)** CASPASE-1, and **(E)** CASPASE-4. Each treatment had three samples (n = 3) and was repeated three times to reduce system errors; “※” means there was a significant difference (*P < 0.05*) compared with negative control (LPS, 0 µg/ml).

Inflammasomes and *GSDMD* mRNA and protein expression level were determined to identify whether LPS leads to inflammation *via* the classical or non-classical inflammasome pathways and/or the occurrence of cellular pyroptosis is mediated through GSDMD. After LPS exposure for 24 h, the 10 and 30 µg/ml treatments significantly increased the mRNA expression of *NLRP3*, *Caspase-1*, *Caspase-4*, and *GSDMD* (**Figures 4A, C–E**) and the protein expression of caspase-1 and caspase-4 (**Figures 5C, D**) compared to controls. The mRNA expression of *ASC*, and the protein expression of NLRP3 and ASC showed no statistically significant differences among the groups (**Figures 4B** and **5A, B**). Cleaved caspase-1 protein could be observed (**Figure 5C**). The data suggest that LPS exposure for 24 h leads to BEEC inflammation *via* classical and non-classical inflammasome pathways.

### LPS Exposure for 24 h Leads to BEEC Inflammation and Cleaved GSDMD to Pyroptosis

**Figure 6:**
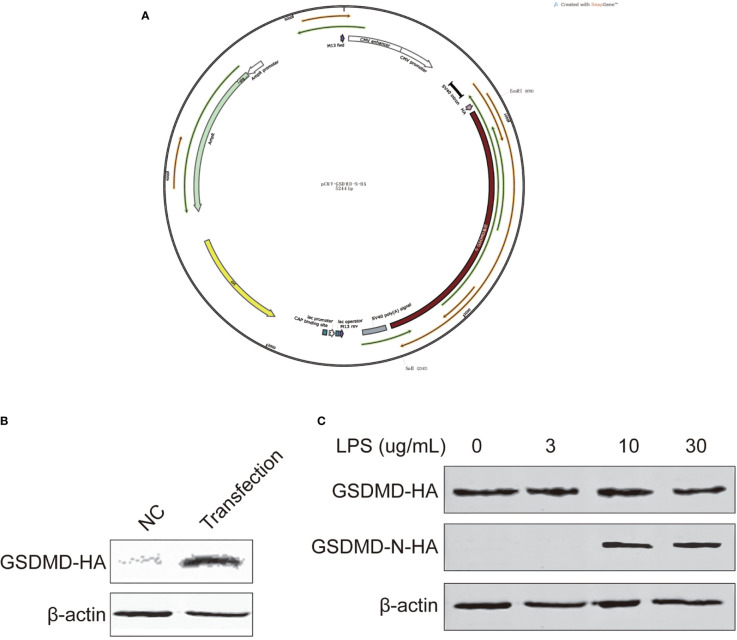
Vector construction, transfection. GSDMD and cleaved N-terminal protein expression of cells treated with different concentrations of LPS for 24 h. **(A)** the vector, **(B)** vector transfection, and **(C)** GSDMD and cleaved N-terminal. Each treatment had three samples (n = 3) and was repeated three times to reduce system errors.

We constructed a pCMV-GSDMD-N-HA vector (**Figure 6A**) and transfected it to BEECs to overexpress GSDMD, and detected protein expression with Western blotting after 48 h to ensure the transfection was successful (**Figure 6B**). The cell inflammation model was rebuilt, total protein was extracted, and GSDMD expression and its cleaved N terminal protein were detected using Western blotting. The results showed that BEEC exposed to LPS (10 and 30 µg/ml) for 24 h could cleave GSDMD and lead to pyroptosis (**Figure 6C**).

## Discussion

The endometrium is the first line of defense for the uterus to prevent infection. When pathogenic bacteria invade during postpartum, the epithelial cells activate the innate immune functions, such as the secretion of antibacterial peptides, acute phase proteins, and Toll-like receptor-related functions. When endometrial inflammation occurs, it significantly affects the reproductive function of the uterus (37–39). Therefore, maintaining the proper functioning of endometrial epithelial cells and a complete endometrial gland plays a crucial role in preventing infection and ensuring an excellent reproductive function. The adoption cell models to study the molecular pathological mechanism BEECs during inflammation can contribute to the further understanding, prevention, and treatment of cow endometritis.

TNF-α, IL-1β, and IL-6 are some of the pro-inflammatory cytokines, which play an important role in the inflammatory response during infectious process (38). TNF-α can initiates cytokine cascades, increased the expression of adhesion factors and vascular permeability (40, 41). IL-1β regulates and activates dendritic cells, macrophages, and neutrophils, which are important for enhancing inflammatory response (42). IL-6 regulates immune responses and acute phase responses and is involved in the anti-infective immune response (43). LPS is widely used to construct models and research into acute lung injury, mastitis, and endometritis (44–46). We used different concentrations of LPS to act on BEECs for 24 h to construct a model of cell inflammation. The levels of TNF-α, IL-1β, and IL-6 cellular secretion and cellular expression of *TNFα*, *IL1B*, and *IL6* mRNA were significantly increased, indicating that the model was successfully constructed. LDH is an enzyme that exists in cells. Only little is secreted when the cell membrane is intact, but it will be released outside of cells in large amounts if the cell membrane is ruptured. Therefore, LDH is a sign of the integrity of the cell membrane, and LDH content is often used to measure cell mortality and cell membrane integrity (47, 48). The scanning electron microscope is a common technique for observing the surface structure of objects, and it is also used to observe the pores in the cell membrane during pyroptosis (25). Our results suggest that cell death may occur *via* the rupture of the cell membrane, a hallmark of pyroptosis.

NLRP3 is a member of the NLR family, and it can receive external stimuli through its C-terminal LRR and combine with the PYD domain of ASC through its N-terminal PYD domain. Then, the protein complex can recruit pro-caspase-1 through the CARD domain of ASC to combine with the CARD domain of pro-caspase-1 and leads pro-caspase-1 to mature by self-cleavage. Then, inflammasome immune complexes are formed and can convert cytokines (e.g., IL-18 and IL-1β) precursors to mature forms and cause pyroptosis (49–51). Our research results show that: after 24 h exposure to different concentrations of LPS, the mRNA and protein expression of NLRP3, ASC, and CASPASE-1 were increased in BEECs, and caspase-1 protein cleavage could be observed, indicating that LPS can induce BEEC pyroptosis through the NLRP3 classical inflammasome pathway.

The non-classical inflammasome pathway is also called the caspase-1-independent inflammasome pathway because the cleavage of GSDMD is not by caspase-1, but *via* caspase-4/5/11, an intracellular LPS receptor. It was previously thought that LPS could only stimulate TLR4 receptors outside the cell and cause inflammation through the NF-κB signaling pathway or the classical inflammasome signaling pathway. However, recent studies have found a direct receptor caspase-4/5/11 for LPS in the cell. Its expression depends on TLR2/3/4 receptor activation, but its activation must involve the participation of intracellular LPS. The activation of caspase-4/5/11 is also different from the self-cleavage of caspase-1, where oligomerization occurs. Studies have shown that the activation of caspase-4/5/11 is dependent on its CARD domain and the hexadecyl lipid A in LPS (52–55), and a research on primary endometrial epithelial and stromal fibroblast cells of bovine have told that: NLRP3 and caspase-4 mediated IL-1β production in a non-canonical inflammasome way (56). Our results show that after BEEC exposure 24 h with different concentrations of LPS, the mRNA and protein expressions of caspase-4 are increased, indicating that LPS could induce BEECs pyroptosis through the non-classical inflammasome pathway.

This study shows that LPS could cause BEEC inflammation and mediate pyroptosis through not only NLRP3 classical but also non-classical inflammasome pathways. LPS could be transferred into the cytoplasm *via* the bacterial outer membrane vesicles (OMV) or the high-mobility group box 1 (HMGB1) protein and receptor for advanced glycation end products (RAGE) complexes to trigger caspase-4/5/11 (57), further research could be required to evaluate the molecular mechanism through which microorganisms penetrate the cell receptors and cause damage to the cell’s cytoplasm leading to pyroptosis.

## Data Availability Statement

The original contributions presented in the study are included in the article/**Supplementary Material**. Further inquiries can be directed to the corresponding author.

## Author Contributions

MY analyzed the experiment data and drafted the manuscript. LJ, SX,YJ, JW, and BI helped to conceptualized the work and collected data. AO, WY, WH, DZ, and YY participated in the design of the study and critically reviewed the manuscript. WY and YT participated in the design of the study and managed the project. All authors contributed to the article and approved the submitted version.

## Funding

This research was funded by the National Key R&D Program of China (No.2017YFD0502201), the Science and Technology Innovation Project (No. CAAS-ASTIP-2014-LIHPS-03), and the Key Research and Development Plan of Gansu province (20YF8NA029), and the Talent innovation and entrepreneurship project of Lanzhou city (2018-RC-91). The funding agencies had no role in the study design, data collection, analysis, or preparation or decision to publish the manuscript.

## Conflict of Interest

The authors declare that the research was conducted in the absence of any commercial or financial relationships that could be construed as a potential conflict of interest.
