# Supplementary material for: LPS Mediates Bovine Endometrial Epithelial Cell Pyroptosis Directly Through Both NLRP3 Classical and Non-Classical Inflammasome Pathways
Source: Front Immunol. 2021 May 28;12:676088. doi: 10.3389/fimmu.2021.676088 (PMC8195237; doi:10.3389/fimmu.2021.676088)
Supplement: Supplementary file 1 [file DataSheet_1.docx]

Supplemental Table 1 RNA quality

| LPS (µg/mL) | A260/280 | Concentration (ng/µL) |
| --- | --- | --- |
| 0  0  0 | 1.829  1.840  1.841 | 588.32  560.44  621.52 |
| 3  3  3 | 1.865  1.811  1.970 | 586.24  564.12  589.40 |
| 10  10  10 | 1.850  1.885  1.922 | 572.40  555.67  614.48 |
| 30  30  30 | 1.861  1.889  1.848 | 578.32  535.28  571.00 |

Supplemental Table 2 Primers used for RT-qPCR

| **Gene** | **Forward(5’-3’)** | **Reverse(5’-3’)** | **Product Size** | **Concentration** |
| --- | --- | --- | --- | --- |
| *ACTB* | CTCTTCCAGCCTTCCTTCCT | GGGCAGTGATCTCTTTCTGC | 178bp | 200nM |
| *IL6* | GCTGAATCTTCCAAAAATGGAGG | GCTTCAGGATCTGGATCAGTG | 200bp | 200nM |
| *IL10* | CCTTGTCGGAAATGATCCAGTTT | TCAGGCCCGTGGTTCTCA | 67bp | 200nM |
| *TNFα* | TCCAGAAGTTGCTTGTGCCT | CAGAGGGCTGTTGATGGAGG | 144bp | 200nM |
| *IL1B* | CCTCGGTTCCATGGGAGATG | AGGCACTGTTCCTCAGCTTC | 119bp | 200nM |
| *IL18* | TCAGATAATGCACCCCAGACC | GATGGTTACGGCCAGACCTC | 78bp | 200nM |
| *NLRP3* | CTTTCTGGACTCTGACCGGG | CTCCCATTCTGGCTCTTCCC | 312bp | 200nM |
| *ASC* | ATGAAGCTGCTTTCAGTGCC | ACAAAGTGCAGTCCTGGCTTG | 272bp | 200nM |
| *Caspase-1* | AATAAATGGCTTGCTGGATGAG | CCTCCTGGTCCTGAAGATGC | 327bp | 200nM |
| *Caspase-4* | TATAAAAGCTCCTGAGGAAACT | TTCCACAGTGTAGCCAAGAC | 268bp | 200nM |
| *GSDMD* | CCATTGGGAGCATGGCCTC | GCGGTCTCCAGAATCGTGAA | 165bp | 200nM |


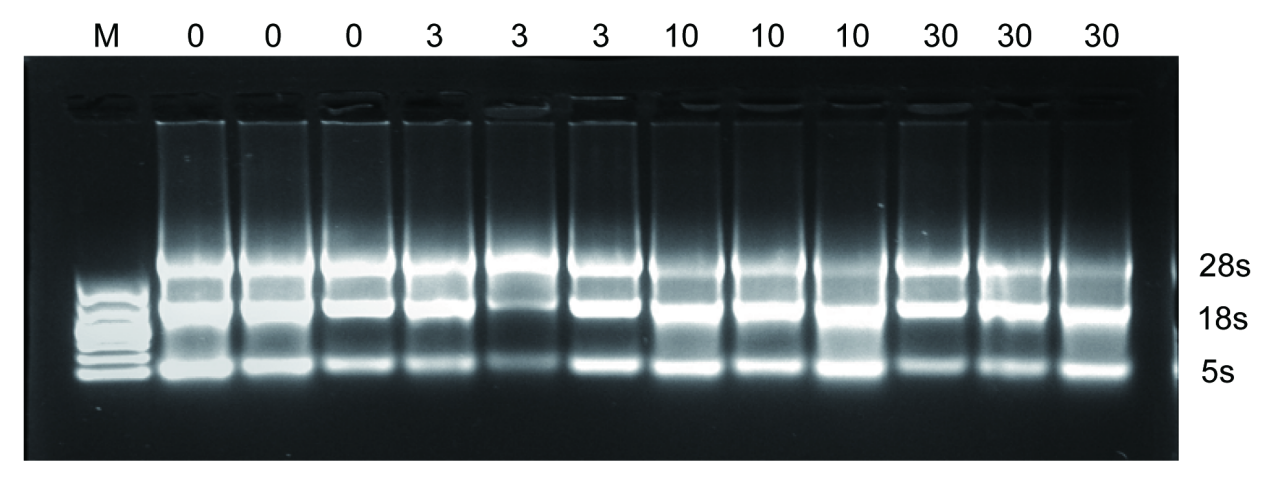
Supplemental Figure 1 RNA Gel Electrophoresis

1. Marker, DL 2000; 0) LPS 0 ug/mL; 3) LPS 3ug/mL; 10) LPS 10ug/mL; 30) LPS 30ug/mL; Each group was repeated three times
